# A Canadian Perspective on the Treatment of Waldenström Macroglobulinemia

**DOI:** 10.3390/curroncol29100560

**Published:** 2022-09-28

**Authors:** Rayan Kaedbey, Nicholas Forward, Laurie H. Sehn, Mona Shafey, Sarah Doucette, Christine I. Chen

**Affiliations:** 1 Department of Hematology, Jewish General Hospital, Montreal, QC H3T 1E2, Canada; 2 Department of Medicine, Dalhousie University/Nova Scotia Health, Halifax, NS B3H 2Y9, Canada; 3 Department of Medical Oncology, BC Cancer Centre for Lymphoid Cancer, Vancouver, BC V5Z 4E6, Canada; 4 Department of Medicine, Division of Hematology, Foothills Medical Centre and University of Calgary, Calgary, AB T2N 2T9, Canada; 5 Impact Medicom Inc., Toronto, ON M6S 3K2, Canada; 6 Department of Medical Oncology and Hematology, Princess Margaret Cancer Centre, 610 University Avenue, Suite 6-225, Toronto, ON M5G 2M9, Canada

**Keywords:** Waldenström macroglobulinemia, chemoimmunotherapy, Bruton’s tyrosine kinase inhibitors

## Abstract

Waldenström macroglobulinemia (WM) is a slowly progressing B-cell non-Hodgkin lymphoma characterized by monoclonal IgM gammopathy in the blood and infiltration of the bone marrow by clonal lymphoplasmacytic cells. As an incurable disease, the goals for therapy for WM are to relieve symptoms, slow disease progression, prevent organ damage, and maintain quality of life. However, given the rarity of WM, clinical trials comparing treatments for WM are limited and there is no definitive standard of care. The selection of first-line WM therapy is thus based on patient factors, disease characteristics, and drug access, with bendamustine-rituximab and Bruton’s tyrosine kinase (BTK) inhibitor therapy considered preferred treatments. Other treatments such as proteasome inhibitor- or purine analogue-based therapy, alternative chemoimmunotherapy, and autologous stem cell transplantation are generally reserved for the relapsed setting but may be used in rare circumstances in earlier lines of therapy. This paper summarizes the efficacy and safety of these WM therapies and discusses considerations for treatment from a Canadian perspective.

## 1. Introduction

Waldenström macroglobulinemia (WM) is a rare, slowly progressing sub-type of B-cell non-Hodgkin lymphoma (NHL) characterized by monoclonal IgM gammopathy in the blood and infiltration of the bone marrow by clonal lymphoplasmacytic cells [1]. The incidence rate for WM is 4–6 cases per million people per year in North America, most commonly in those over the age of 65 years [2,3]. The 5-year relative survival rate for WM is approximately 78%, with prognosis varying by risk categories based on the International Prognostic Score System for WM [4,5,6]. A trend for improved survival in WM has been observed in recent decades, which likely reflects an improvement in WM therapies [7].

Waldenström macroglobulinemia remains an incurable disease, therefore the goals of therapy are to relieve symptoms, slow disease progression, prevent organ damage, and maintain a high quality of life. Therapy selection is complicated in WM as there is no definitive standard of care [8]. This is due to published trials on WM predominantly being single-arm, phase 2 studies that combine both treatment-naive and relapsed WM or are phase 3 studies evaluating a combination of B-cell NHLs. Thus, treatment selection for WM must be made based on patient, disease, and other factors. This paper aims to summarize the currently available treatments for WM in Canada and discuss considerations for treatment selection.

## 2. Clinical Presentation, Diagnosis, and Initiation of Treatment

Diagnosis of WM requires histopathological confirmation of ≥10% bone marrow infiltration by lymphoplasmacytic cells and the detection of IgM protein. The typical immunophenotypic profile of WM cells include surface expression of IgM, CD19, CD20, CD22, FMC7, CD25, and CD27 [9,10]. Waldenstrom Macroglobulinemia is generally distinguished from other B-cell malignancies by lack of expression of CD5, CD10, CD11c, CD23, and CD103, CD138; however, these markers are expressed in a portion of WM cases, presenting challenges to diagnosis [9,10,11].

In over 90% of WM cases, malignant B cells are found to harbour alterations in the *MYD88* gene, most frequently resulting in an amino acid substitution from leucine to proline at codon 265 (*MYD88*^L265P^) [12,13,14]. Identification of *MYD88*^L265P^ mutations can help to discriminate WM from other B-cell malignancies with similar clinicopathological features but where *MYD88* mutations occur infrequently [15]. Mutations in the *CXCR4* gene are also distinctive to WM and are observed in approximately 40% of cases; almost always co-occurring with *MYD88*^L265P^ [16]. Together these mutations may partly explain the biological heterogeneity of WM and have been associated with variance in prognosis and response to therapy [15,17].

About one quarter of patients who meet the diagnostic criteria for WM are asymptomatic and are considered to have smoldering WM [18]. These patients do not require immediate treatment, as studies have shown that postponing treatment until symptoms develop has no significant effect on survival outcome, similar to other types of indolent B-cell NHL [19,20]. However, they should be closely monitored, particularly in the first 5 years of diagnosis as the rate of progression to symptomatic WM is highest during this time (approximately 10–12% per year) and then tapers off [20,21]. Factors associated with a higher risk of progression include IgM levels 45 g/L or greater, bone marrow lymphoplasmacytic infiltration 70% or greater, β2-microglobulin 4.0 mg/L or greater, and albumin 35 g/L or less and may be used to prompt closer monitoring [21].

Most patients with WM will present with or progress to disease that requires therapy initiation [7,20,21]. Therapy is indicated for patients with B symptoms (e.g., unexplained fever or weight loss, night sweats, fatigue), cytopenias related to bone marrow involvement (hemoglobin < 10 g/dL or platelets < 100,000/μL), organ infiltration leading to splenomegaly and lymphadenopathy, and presence of IgM-mediated complications including hyperviscosity, neuropathy, cryoglobulinemia, cold aglutinin disease, and amyloidosis [1].

Symptomatic hyperviscosity, which is observed in approximately one third of patients, can manifest as blurring/loss of vision, nystagmus, headache, dizziness, and mucocutaneous bleeding [9]. Peripheral neuropathy may present in about one quarter of patients at diagnosis as a result of hyperviscosity, infiltration of malignant cells to the nervous system, binding of IgM antibodies to myelin-associated glycoprotein in the nerves, or IgM protein deposits from cryoglobulinemia and amyloidosis [22]. Cryoglobulinemia and cold agglutinin disease can both manifest as purpura, Raynaud’s syndrome, and acrocyanosis in response to cold, with cryoglobulin deposits also able to impact organ function (notably the kidney) [23]. The manifestations of amyloidosis are diverse, including renal, hepatic, and cardiac dysfunction which may be similar to manifestations of other common conditions in the elderly. Suspicion of any IgM-mediated complication should prompt diagnostic confirmation (e.g., fundoscopic examination, serum viscosity, cryoglobulin and cold agglutinin titer, evaluation of fat or bone marrow biopsy with Congo red staining) as this would indicate a need for treatment that can quickly reduce IgM levels.

## 3. Treatment Options for Waldenström Macroglobulinemia

### 3.1. Chemoimmunotherapy (CIT)

Chemotherapy combined with the anti-CD20 monoclonal antibody rituximab is widely used to treat WM. The only CIT to be evaluated in a phase 3 randomized controlled trial for the first-line treatment of WM is bendamustine-rituximab (BR, Table 1). The StiL NHL1-2003 trial investigated BR versus rituximab-cyclophosphamide-doxorubicin-vincristine-prednisolone (R-CHOP) in a non-inferiority trial in patients with various indolent lymphomas [24]. Among the 41 patients with WM in the trial (22 patients in the BR arm, 19 patients in the R-CHOP arm), progression-free survival (PFS) for BR was non-inferior to R-CHOP (median PFS: 69.5 months vs. 28.1 months; hazard ratio [HR], 0.33, 95% confidence interval [CI] 0.11–0.64; *p* = 0.0033). In the StiL NHL7-2008 trial, which examined the role of rituximab maintenance in 218 patients with WM following response to BR induction, the median PFS with or without rituximab maintenance exceeded 6 years, reaffirming the impressive efficacy of this regimen [25]. This study also found that patients who do not respond to BR or progress within 24 months have a markedly poorer outcome and should be identified as a high-risk group that would benefit from novel therapies. Retrospective studies of BR in patients with relapsed/refractory WM suggest it is an effective regimen in this setting, achieving overall response rates (ORRs) over 80% and median PFS beyond 2 years [26,27]. Overall, BR has demonstrated rapid onset of action in WM, with responses typically seen in the first 1–2 cycles [24,25,26,27].

Prior to publication of the StiL NHL1 data, rituximab, cyclophosphamide, and dexamethasone (R-CD) was frequently used in Canada for the treatment of WM. In a multicentre phase 2 study evaluating R-CD in treatment-naïve WM, 83% of patients achieved response and the median PFS was 35 months (95% CI 22–48) [28,29]. The overall survival (OS) rate at 8 years was 47%, albeit, 43% of deaths were deemed unrelated to WM. The toxicity data for R-CD was favourable, with only 9% and 13% of patients experiencing grade 3/4 neutropenia and infection, respectively. Although there are no prospective randomized studies comparing BR with R-CD for the first-line treatment of WM, two retrospective studies have suggested that BR is associated with increased activity compared to R-CD, but at the expense of increased toxicity [30,31]. Time to response also appears to be prolonged with R-CD compared with BR [30].

Other cyclophosphamide-based therapies including cyclophosphamide and rituximab combined with prednisone (R-CP), vincristine and prednisone (R-CVP), or vincristine, doxorubicin, and prednisone (R-CHOP) have demonstrated efficacy in WM, with ORRs of approximately 90–95% being achieved in clinical studies [37]. In terms of toxicity, neutropenic fever and peripheral neuropathy occur at significantly higher rates with R-CVP and R-CHOP than R-CP [37]. In addition, R-CHOP demonstrated a higher rate of cytopenias, infections, and alopecia compared with BR in the STiL NHL1-2003 trial [24].

Purine analogues (e.g., fludarabine or cladribine) in combination with rituximab have demonstrated efficacy in single-arm clinical trials of patients with treatment-naïve WM, with ORRs between 80–95% and major response rates between 75–90% [33,34,35,36]. Impressive response durations of over 5 years can be achieved; however, these chemotherapy regimens are associated with long-term cytopenia and secondary malignancies, thus are used less frequently in current-day treatment.

Rituximab monotherapy has also been studied in WM, with clinical trials reporting ORRs at or below 50% and median PFS under 24 months [38,39,40,41]. Due to the moderate activity and relatively short durations of response, rituximab monotherapy is typically reserved for select frail patients or those mainly experiencing neuropathy without other disease burdens such as ctyopenias, splenomagaly, or lymphadenopathy. As rituximab monotherapy is associated with a transient increase in IgM levels, it is not appropriate for patients with hyperviscosity [42]. When rituximab is given in combination with other agents, IgM flares are less common.

The benefit of rituximab maintenance following response to BR is unclear given that the StiL NHL7-2008 trial did not demonstrate a survival benefit for maintenance rituximab compared with observation in patients with WM. The median PFS was numerically longer in the rituximab maintenance arm (101 vs. 83 months), but the difference was not statistically significant (HR 0.80; 95% CI 0.51–1.25, *p* = 0.32) [25]. Based on this study, rituximab maintenance is not routinely used in Canada; however, it may be discussed with the patient while acknowledging the StiL NHL7-2008 data. The potential for severe outcomes following SARS-CoV-2 infection in individuals receiving rituximab therapy should also be discussed [43]. Rituximab maintenance may be useful in certain circumstances, such as in patients who received BR induction without at least a PR or in patients who received alternative CIT, as the benefit of rituximab maintenance has not been evaluated in these populations.

### 3.2. Proteasome Inhibitor-Based Therapy

Proteasome inhibitors have demonstrated efficacy in a number of B-cell malignancies, including WM (Table 2). Bortezomib is the first proteasome inhibitor to be examined in WM. It was initially studied in the relapsed/refractory setting as a twice-weekly, single-agent intravenous therapy, which achieved ORRs of 26–85% [44,45]. However, this dosing schedule led to frequent high grade peripheral neuropathy (approximately 20% of patients with grade ≥ 3). Bortezomib given weekly in combination with rituximab has shown similar efficacy in clinical trials, with fewer cases of high-grade neuropathy [46]. Globally, bortezomib has transitioned from intravenous to subcutaneous delivery which has also contributed to a decrease in neuropathy rates [47].

Phase 2 trials have evaluated both a twice-weekly and once-weekly bortezomib regimen, given in combination with dexamethasone and rituximab (BDR), in patients with treatment-naïve WM. Both regimens produced ORRs above 80%, with the once-weekly regimen again resulting in fewer cases of grade ≥3 peripheral neuropathy [51,55]. Bortezomib given with dexamethasone, cyclophosphamide, and rituximab has also demonstrated a high rate and depth of response in a phase 2 randomized trial of patients with WM; however, PFS was not found to be significantly prolonged over R-CD alone [49]. Bortezomib, cyclophosphamide, and rituximab (BCR) has also demonstrated similar overall and major response rates to FCR in a phase 2 in treatment-naïve WM and BCR resulted in less hematological toxicities [56].

Second-generation proteasome inhibitors, including carfilzomib and ixazomib, have been evaluated in WM and are associated with lower neuropathic risk. Phase 2 studies evaluating carfilzomib or ixazomib in combination with rituximab have reported ORRs of 71–96% in treatment-naïve and previously treated patients with WM [52,53,54] (Table 2). Hyperglycemia and infusion-related reactions were the most common grade 3/4 adverse events in these studies.

### 3.3. Bruton’s Tyrosine Kinase (BTK) Inhibitors

Prospective studies have demonstrated high response rates and sustained remissions with BTK inhibitors in WM (Table 3). Both ibrutinib and zanubrutinib are covalent-binding BTK inhibitors which are approved by Health Canada for the treatment of patients with previously untreated or relapsed WM. Evidence for the efficacy of ibrutinib monotherapy in treatment-naïve WM comes from a small, single-arm, prospective trial of 30 patients [57]. In this study, at a median follow-up of 50 months, major response rate was 87% and the 4-year PFS rate was 76%. Similar results were demonstrated in a single-arm trial of ibrutinib in patients with relapsed WM [58].

Ibrutinib has also been approved by Health Canada in combination with rituximab based on the phase 3 iNNOVATE trial, which found that compared to placebo-rituximab, treatment with ibrutinib-rituximab led to higher rates of major response (76% vs. 31%) and improved PFS (54-month PFS rate: 68% vs. 25%; HR 0.25, 95% CI 0.15–0.42) [40,59]. However, given the excellent response rates and PFS that can be achieved with ibrutinib monotherapy and the fact that clinical trials in CLL have demonstrated that there is no PFS benefit for ibrutinib-rituximab versus ibrutinib monotherapy, ibrutinib is generally given as monotherapy in Canada [60]. Ibrutinib-rituximab was associated with higher rates of atrial fibrillation (12% vs. 1%) and hypertension (13% vs. 4%) and lower rates of infusion reactions (1% vs. 16%), and IgM flare (8% vs. 47%) compared to rituximab-placebo [59].

Zanubrutinib monotherapy is indicated by Health Canada for the treatment of WM at a recommended total daily dose of 320 mg given either once- or twice-daily. However, twice-daily dosing was shown to achieve optimal BTK receptor occupancy in a dose finding study [70]. Approval of zanubrutinib is based on the phase 3 ASPEN trial, which randomized 201 patients with MYD88 mutated WM to treatment with twice-daily zanubrutinib or once-daily ibrutinib, until disease progression or unacceptable toxicity [66]. In an updated report of ASPEN, at a median follow-up of 43 months, zanubrutinib continued to demonstrate trends for more favourable very good partial response rates (36.3% vs. 25.0%) and PFS (42-month PFS rate: 78.3% vs. 69.7%); however, the results were not statistically significant [65]. Estimated 42-month OS rates were similar between arms. Adverse events reported less frequently for zanubrutinib versus ibrutinib, included atrial fibrillation/flutter (7.9% vs. 23.5%), diarrhea (21.8% vs. 34.7%), muscle spasms (10.9% vs. 28.6%), hypertension (14.9% vs. 25.5%), and pneumonia (5.0% vs. 18.4%). While there was a higher rate of neutropenia with zanubrutinib (33.7% vs. 19.4%), infection rates were similar between arms.

Other BTK inhibitors not currently indicated in Canada for the treatment of WM have also demonstrated efficacy and safety in clinical trials including acalabrutinib, tirabrutinib, and the non-covalent binding pirtobrutinib (loxo-305) [67,68,69].

### 3.4. Stem Cell Transplantation

Several case series have reported on the efficacy of autologous stem cell transplantation (ASCT) for patients with treatment-naïve WM [71,72]; however, few patients are eligible to receive this therapy due to advanced age and comorbidities. Transplant is generally considered an option in the relapsed setting as there are no trials comparing ASCT to current first-line agents for WM. The European Society for Blood and Marrow Transplantation retrospectively analyzed the outcomes of 615 patients undergoing ASCT for WM [73]. They reported a 5-year OS and disease-free survival rate of 65% and 46%, respectively, and a non-relapse mortality rate of 7%. Allogeneic stem cell transplantation for WM has been reported in small case series as a treatment that can prolong PFS and OS in some highly selected patients; however, it is not commonly recommended due to its high rate of non-relapse mortality [74,75].

### 3.5. Other Novel Therapies under Investigation

Several other therapies are currently being investigated in clinical trials for WM, some of which have reported encouraging results [76]. Venetoclax, a first-in-class, selective B-cell lymphoma-2 (BCL-2) inhibitor demonstrated a major response rate of 84% and median PFS of 30 months in a phase 2 study of 32 patients with previously treated WM (16 of which received prior BTK inhibitor therapy) [77]. Studies evaluating venetoclax in combination with rituximab or ibrutinib for the treatment of WM are ongoing (NCT05099471, NCT04273139).

T-cell-based immunotherapies are also being explored, including chimeric antigen receptor T cell (CAR T) therapy and bispecific T-cell engagers (BiTEs). Preliminary, single-centre studies of CD20 and CD19 directed CAR T therapy in B-cell malignancies have demonstrated high response rates for patients with WM [78,79]. Multicentre phase 1/2 studies for these therapies are underway (NCT05360238, NCT04450069). Studies have also demonstrated proof-of-concept support for the use of CD19/CD3 and CD20/CD3 BiTE immunotherapy in B-cell NHL [80,81]. A phase 1 study of the anti-CD20/anti-CD3 BiTE, plamotamab (XmAb13676), in B-cell malignancies is ongoing (NCT02924402).

## 4. Canadian Perspective on Treatment Selection

### 4.1. Treatment Selection in First Line

**Figure 1:**
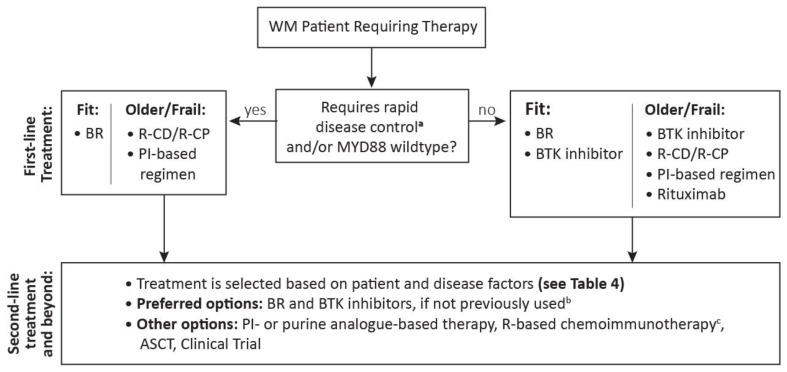
Treatment algorithm for symptomatic Waldenström macroglobulinemia requiring treatment initiation (cf. Table 4). ^a^ Plasmapheresis is indicated in patients with symptomatic hyperviscocity. Prophylactic plasmapheresis may be considered in patients who have asymptomatic elevated IgM, cryoglobulinemia, cold agglutinin disease, or IgM mediated neuropathy. ^b^ BR may be repeated if patients remained progression-free for 2–3 years after response to first-line BR ^c^ Exclude rituximab if progressed <12 months on prior rituximab-based therapy. ASCT, autologous stem cell transplant; BR, bendamustine-rituximab; BTK, Bruton’s Tyrosine Kinase; PI, proteasome inhibitor; R, rituximab; R-CD, rituximab-cyclophosphamide-dexamethasone; R-CP, rituximab-cyclophosphamide-prednisone; WM, Waldenström macroglobulinemia.

Selection of first-line treatment for WM is based on patient factors, including age, comorbidities, and preferences, as well as disease factors, including disease-related complications and mutation profile (Figure 1). American and European practice guidelines recommend BR, BTK inhibitors, and BDR as preferred first-line treatment for WM [8,10]. Canadian practice is generally aligned with these recommendations as BR and BTK inhibitors are considered standard of care for first-line therapy; however, bortezomib is generally reserved for the relapsed setting due in part to the incidence of neuropathy, as well to restricted access in some provinces, given the lack of Health Canada indication in WM. Bendamustine-rituximab is a favourable treatment for patients who are younger and fit, who may be better able to tolerate hematological toxicity. BR has a rapid onset of action and therefore may be useful in highly symptomatic patients or those with high IgM levels, requiring rapid responses; however, it is important to consider the increased risk of myelodysplastic syndrome. Bendamustine-rituximab may also be selected for patients who prefer a time-limited therapy and can travel to the treatment centre for infusions.

BTK inhibitors are associated with less toxicity than BR and are thus more suitable for older patients with comorbidities. As zanubrutinib demonstrated similar efficacy but better tolerability than ibrutinib in the ASPEN trial [65], it is now generally preferred over ibrutinib, particularly for patients with cardiovascular comorbidities. Selection of either ibrutinib or zanubrutinib may also depend on ease of access to each agent. As of September 2022, ibrutinib is accessible through public reimbursement for the first-line treatment of WM in some provinces, while zanubrutinib is not yet reimbursed in the first-line but is accessible by compassionate access across Canada. For patients who are already taking ibrutinib but experience intolerable adverse events, there is evidence that switching to zanubrutinib is safe and effective at prolonging PFS [82]. BTK inhibitors may also be favoured for patients who prefer the ease of administration of oral therapies, who have difficulty travelling to treatment centres for infusions, or who are concerned about long-term toxicities associated with CIT.

Bortezomib is occasionally used in the first-line setting to treat patients with renal dysfunction or who are taking concomitant medications, such as blood thinners, that may increase the risk of BTK inhibitor-induced bleeding. Bortezomib also has a rapid onset of action without causing IgM flare, and therefore may be useful in patients with hyperviscosity.

Other therapies that are less frequently used for the first-line treatment of WM include cyclophosphamide-based regimens, which are less effective than other CIT but are a more tolerable option for frail patients who are also looking for finite treatment or who cannot access BTK inhibitors in the first line. Rituximab monotherapy is rarely used given the number of effective treatment options available for WM and the risk of IgM flare; however, it may be used for frail patients in some circumstances.

### 4.2. Treatment Selection by MYD88 and CXCR4 Mutation Status

Clinical trials are beginning to analyze outcomes by *MYD88* and *CXCR4* mutation status, although reported samples sizes are small. In the ibrutinib monotherapy trials, responses appeared to be more frequent and deeper among those with *MYD88^L265P^* mutations and wild-type CXCR4 [57,62]. Among 11 patients with wild-type *MYD88* across these two trials, only 2 patients achieved a major response and PFS was markedly lower than in patients with *MYD88^L265P^* mutations. Given the modest activity of ibrutinib in patients with *MYD88* wild-type WM, a second, single-arm cohort of the ASPEN trial enrolled patients with *MYD88* wild-type (*n* = 26) or unknown (*n* = 2) status to receive zanubrutinib monotherapy [83,84]. In this cohort, major response rates improved over time from 54% at a median follow-up of 17.9 months to 65% at a median follow-up of 42.9 months [65], suggesting some activity for zanubrutinib in patients with MYD88 wild-type, albeit at a slower rate. A larger study of zanubrutinib in patients with *MYD88* wild-type is needed to confirm this signal. The activity of BR and R-CD appears to be unaffected by *MYD88* mutation status, based on a retrospective study of 48 patients (10 patients with wild-type *MYD88*) receiving these therapies in clinical practice [31].

Mutations in *CXCR4* appear to negatively impact efficacy with ibrutinib, with lower major response rates, longer time to response, and poorer PFS observed compared to *MYD88^L265P^/CXCR4* wild-type patients [57,62]. A similar decrease in very good partial response for patients with *CXCR4* mutations compared to those without mutations was observed for zanubrutinib and ibrutinib in the ASPEN trial [66]. An exploratory analysis of ASPEN observed deeper and faster responses with zanubrutinib versus ibrutinib in patients with *CXCR4* mutations, although the study was not powered to examine a significant difference in response in this subgroup of patients [65]. In small phase 2 trials of carfilzomib- and ixazomib-based therapies, ORRs, major response rates, and PFS were similar between patients with and without *CXCR4* mutations; however, responses were deeper and achieved faster in those patients without *CXCR4* mutations [53,85]. The activity of BR also does not appear to be impacted by *CXCR4* mutation status [86].

The current data support prioritization of CIT regimens and proteasome inhibitors for the first-line treatment of patients with wild-type *MYD88* WM. Testing for CXCR4 mutation status is variable across Canada and presently does not guide treatment decisions. However, for those who can access testing, knowing whether a patient has a CXCR4 mutation can help to anticipate a delayed response to BTK inhibitor therapy. The National Comprehensive Cancer Network guidelines for WM recommend all patients who are being considered for BTK inhibitor therapy be tested for *CXCR4* mutations as certain mutations have been associated with ibrutinib resistance [8].

### 4.3. Management of Patients with Elevated IgM Levels

Special management considerations are needed in patients with high baseline IgM levels (over 50–60 g/L). For elevated IgM resulting in hyperviscosity symptoms, plasmapheresis is recommended. For patients with elevated IgM who are asymptomatic, the optimal management approach is unclear. Prophylactic plasmapheresis may be considered in these patients or treatment may be initiated followed by close monitoring of IgM levels over several weeks. When rituximab-based therapies such as BR are used, it may be reasonable to hold rituximab treatment in the first 1 or 2 cycles to reduce the risk of IgM flares; however, it is important to note that in Canada compensatory doses of rituximab following BR induction may not be accessible.

### 4.4. Treatment Selection for Disease-Related Complications

Complications from WM related to high IgM levels such as hyperviscosity as well as other IgM paraprotein mediated complications like neuropathy, amyloidosis, cryoglobulinemia, and cold agglutinin disease require immediate reduction in IgM levels. As BR is associated with deep, durable, and quick responses, it is ideal for patients with IgM complications. In older patients who may not be able to tolerate BR, proteasome inhibitor therapy may be a suitable treatment option in patients who are not presenting with neuropathies as they appear to induce faster responses than BTK inhibitors. In patients with IgM-related neuropathy who are unsuitable for BR, second generation proteasome inhibitors with less neuropathic risk, such as carfilzomib, may be used if accessible.

Bing-Neel syndrome is a rare complication of WM whereby malignant cells infiltrate the central nervous system (CNS) causing neurological disturbances. Treatment should consist of agents known to penetrate the blood–brain barrier, including fludarabine- and cladribine-based regimens, or more recently, BTK inhibitors. There is evidence that BTK inhibitors can pass the blood–brain barrier and effectively manage patients with Bing-Neel syndrome. In a study of 28 patients with WM and Bing-Neel syndrome receiving oral ibrutinib, 85% of patients had symptomatic improvement, 83% had radiologic improvement, and 47% had cleared the disease in cerebrospinal fluid at best response [87]. Zanubrutinib has also demonstrated efficacy in case reports of B-cell malignancies with CNS involvement, including WM, and can be considered for patients with CNS involvement [88,89,90,91,92,93].

### 4.5. Treatment Selection for Relapsed Disease

The same considerations of patient and disease factors apply to treatment selection in the relapsed setting. There is a preference for giving BR or BTK inhibitors in the second line, using whichever agent was not used in the first line. It is possible to retreat patients with BR if they achieved a PFS beyond 2 years after first-line therapy; however, this is less commonly done given the availability of other effective second-line therapies. Bortezomib is generally used in the relapsed setting only after eligible patients have received BR or BTK inhibitors. This is partly due to the incidence of neuropathy, as well to restricted access, given the lack of Health Canada indication in WM.

Stem cell transplantation remains an option in the relapsed setting, with use varying by centre. Patients who have relapsed following CIT and BTK inhibitor therapy may also be encouraged to enroll in a clinical trial where novel therapies can be accessed.

## 5. Summary and Conclusions

Bendamustine-rituximab and BTK inhibitors are currently the most commonly used regimens for treatment of WM in the first- or second-line setting in Canada. Other treatment options, including proteasome inhibitors, cyclophosphamide-based CIT, and purine analogues have also demonstrated activity in WM and are most commonly used in the relapsed setting where they can offer a different mechanism of action for controlling WM. Each treatment for WM has advantages and disadvantages in terms of speed of action, toxicity profile, and drug administration which allows physicians to individualize therapy for their patients (Table 4). However, treatment selection may be limited by disparities in drug access across Canada. Genomic biomarkers, such as *MYD88* and *CXCR4* mutation status, will likely become more influential for treatment decisions in Canada in the future, granted that access to molecular testing is increased. Despite a number of treatment options available and the improved survival observed for WM over the years, a high unmet need remains for patients who respond poorly to available treatments. A better understanding of WM biology and mechanisms of treatment resistance is thus needed to drive the development of novel therapies for these patients.

## Figures and Tables

**Table 1:** Notable clinical trials evaluating chemoimmunotherapy in Waldenström Macroglobulinemia.

Regimen	Phase	Population	ORR (%)	MRR (%)	PFS	Notable Adverse Events
BR vs. R-CHOP [24]	3	TN			Median:	**More common with BR:** rash **More common with R-CHOP:** Alopecia, cytopenia, peripheral neuropathy, stomatitis
		N_(BR)_ = 22	93	NR	70 months	
		N_(R-CHOP)_ = 19	91	NR	28 months	
R-CD [29]	2	TN, N = 72	83	74	2 y: 67%	Neutropenia, IRR, infections
Chlorambucil vs. Fludarabine [32]	3	TN			Median:	**More common with CLB:** second malignancy **More common with F:** Grade 3/4 neutropenia
		N_(CLB)_ = 169	36	NR	27 months	
		N_(F)_ = 170	46	NR	36 months	
Cladribine + rituximab [33]	2	N_(TN)_ = 16	94	79	NR	Neutropenia, cardiac toxicity
		N_(R/R)_ = 13	85			
Fludarabine + rituximab [34]	2	N_(TN)_ = 27	96	89	4 y: 67%	Cytopenia, infection, transformation to aggressive lymphomas
		N_(R/R)_ = 16	94	81	4 y: 38%	
FCR [35]	2	N_(TN)_ = 25 N_(R/R)_ = 57	88 84	64 65	3 y: 96% 3 y: 73%	Cytopenia, infection, MDS
FCR [36]	2	N_(TN)_ = 28	79	74	NR	Neutropenia, MDS
		N_(R/R)_ = 15				

BR, bendamustine-rituximab; CLB, chlorambucil; F, fludarabine; FCR, fludarabine-cyclophosphamide-rituximab; IRR, infusion related reactions; MDS, myelodysplastic syndrome; MRR, major response rate; NR, not reported; ORR, overall response rate; PFS, progression-free survival; R-CD, rituximab-cyclophosphamide-dexamethasone; R-CHOP, rituximab-cyclophosphamide-vincristine-doxorubicin-prednisone; R/R, relapsed/refractory; TN, treatment-naïve; y, year.

**Table 2:** Notable clinical trials evaluating proteasome inhibitors in Waldenström Macroglobulinemia.

Regimen	Phase	Population	ORR (%)	MRR (%)	PFS	Notable Adverse Events
Bortezomib ^a^ [44]	2	N(TN) = 12	25	26	Median:	Peripheral neuropathy, hematologic toxicity
		N(R/R) = 15	27		16 months	
Bortezomib ^b^ [45]	2	N(TN) = 1	85	48	Median:	Neuropathy, hematologic toxicity, dizziness
		N(R/R) = 26			8 months	
Bortezomib ^c^-rituximab [48]	2	TN	92	62	NR	Neuropathy ^j^, Cytopenia, fatigue
		N = 26				
Bortezomib ^c^-rituximab [46]	2	R/R	81	51	Median:	Neuropathy ^j^, Cytopenia
		N = 37			16 months	
Bortezomib ^d^-R-CD vs. R-CD [49]	2	TN				Neuropathy (bortezomib arm) ^j^, Neutropenia (both arms)
		N(B-R-CD) = 101	91	79	2 y: 81%	
		N(R-CD) = 101	87	69	2 y: 73%	
Bortezomib ^e^-rituximab-dexamethasone [50]	2	TN	85	68	Median:	Peripheral neuropathy
		N = 59			43 months	
Bortezomib ^f^-rituximab-dexamethasone [51]	2	TN	96	83	NR	Peripheral neuropathy, neutropenia
		N = 23				
Carfilzomib ^g^-rituximab-dexamethasone [52]	2	TN and R/R	80	71	Median:	Lipase elevation, neutropenia, cardiomyopathy
		N = 31			46 months	
Ixazomib ^h^-rituximab-dexamethasone [53]	2	TN	96	77	Median:	Infection, hyperglycemia, infusion reaction
		N = 26			40 months	
Ixazomib ^i^-rituximab-dexamethasone [54]	1/2	R/R	88	74	NR	Infection
		N = 59				

MRR, major response rate; NR, not reported; ORR, overall response rate; PFS, progression-free survival; R-CD, rituximab-cyclophosphamide-dexamethasone; R/R, relapsed/refractory; TN, treatment-naïve; y, year ^a^ Bortezomib IV at 1.3 mg/m^2^/d on days 1, 4, 8, and 11 in a 21-day cycle, given until PD or until two cycles after CR. ^b^ Bortezomib IV at 1.3 mg/m^2^ on days 1, 4, 8, and 11 up to eight cycles ^c^ Bortezomib IV weekly at 1.6 mg/m^2^ on days 1, 8, 15, every 28 days up to 6 cycles ^d^ Bortezomib SC at 1.6 mg/m^2^ on days 1, 8, 15, every 28 days up to 6 cycles. ^e^ Bortezomib IV at 1.3 mg/m^2^ IV on days 1, 4, 8, and 11 on first 21-day cycle; followed by Bortezomib IV weekly at 1.6 mg/m^2^ on days 1, 8, 15, and 22 every 35 days up to 4 cycles. ^f^ Bortezomib IV at 1.3 mg/m^2^ days 1, 4, 8, and 11 for 4 cycles, followed by 4 maintenance cycles 3 months apart for induction therapy and then four more cycles, each given 3 months apart, for maintenance therapy. ^g^ Carfilzomib IV at 20 mg/m^2^ (cycle 1) and 36 mg/m^2^ (cycle 2 and beyond) on days 1, 2, 8, and 9, every 21 days for 6 cycles, followed by maintenance on days 1, 2 every 8 weeks for 8 cycles. ^h^ Ixazomib orally at 4 mg on days 1, 8, and 15 every 28 days for 6 cycles, followed by 6 maintenance cycles every 8 weeks. ^i^ Ixazomib orally at 4 mg on days 1, 8, and 15 every 28 days for 8 cycles. ^j^ Grade 3/4 peripheral neuropathy occurred in ≤5% of patients.

**Table 3:** Notable clinical trials evaluating Bruton tyrosine kinase inhibitors in Waldenström Macroglobulinemia.

Regimen	Phase	Population	ORR (%)	MRR (%)	PFS	Notable Adverse Events
Ibrutinib [61,62]	2	R/R	91	79	5 y: 54%	Afib
		N = 63				
Ibrutinib [63]	3 ^a^	R/R	87	77	5 y: 40%	Neutropenia, infection, hypertension
		n = 31				
Ibrutinib [57,58]	2	TN	100	87	4 y: 76%	Afib, hypertension
		N = 30				
Ibrutinib-rituximab vs. Placebo-rituximab [40,59]	3	N(TN) = 68	I-R: 92 P-R: 44	I-R: 76 P-R: 31	54 m: I-R: 68% P-R: 25%	**More common with I-R:** Afib, hypertension **More common with P-R:** IRR, IgM flare
		N(R/R) = 82				
Zanubrutinib [64]	2	N(TN) = 24	100	88	2 y: 92%	Neutropenia
		N(R/R) = 53	94	80	2 y: 76%	
Zanubrutinib vs. ibrutinib [65,66]	3				42 m:	**More common with Z:** neutropenia **More common with I:** Diarrhea, muscle spasms, Afib, pneumonia
		N(TN) = 37	Z: 94	Z: 77	Z: 78%	
		N(R/R) = 164	I: 93	I: 78	I: 70%	
Acalabrutinib [67]	2	N(TN) = 14	93	79	2 y: 90%	Headache, infection, neutropenia
		N(R/R) = 92	93	80	2 y: 82%	
Tirabrutinib [68]	2	N(TN) = 18	94	89	NR	Rash, Neutropenia
		N(R/R) = 9	100	89		
Pirtobrutinib [69]	1/2	R/R	68	47	NR	Neutropenia
		N = 26				

^a^ A open-label, single-arm substudy of the phase 3 iNNOVATE trial. Afib, atrial fibrillation; I, ibrutinib; IRR, infusion related reaction; m, month; MRR, major response rate; NR, not reported; ORR, overall response rate; P, placebo; PFS, progression-free survival; R, rituximab; R/R, relapsed/refractory; TN, treatment-naïve; y, year; Z, zanubrutinib.

**Table 4:** Treatment considerations for Waldenström Macroglobulinemia.

Regimen	Advantages	Disadvantages
Preferred regimens		
Bendamustine-rituximab	Ideal for patients who are: ◦ Fit ◦ Require rapid IgM reduction ◦ Have MYD88 wild-type or CXCR4 mutations ◦ Prefer time-limited therapy	Increased risk of: ◦ Cytopenia ◦ Myelodysplasia ◦ Secondary myeloid neoplasm
Zanubrutinib	Ideal for patients who are: ◦ Older/Frail ^a^ ◦ Prefer/require oral therapy Improved safety profile over ibrutinib May be used in patients with CNS disease Once or twice daily dosing may be used ^c^	Slower response than with CIT and proteasome inhibitors Increased risk of neutropenia vs. ibrutinib Access may vary by province ^d^
Ibrutinib +/− rituximab ^b^	Ideal for patients who are: ◦ Older/Frail ^a^ ◦ Prefer/require oral therapy ◦ Do not have access to zanubrutinib May be used in patients with CNS disease	Increased risk of Afib, hypertension, and bleeding vs. zanubrutinib Slower response than with CIT and proteasome inhibitors, particularly in those with CXCR4 mutation Minimal response in MYD88 wild-type Access varies by province
Other regimens		
Rituximab-cyclophosphamide-dexamethasone (or prednisone)	Ideal for patients who are: ◦ Older/unfit ^a^ for BR ◦ Require rapid disease control ◦ Prefer time-limited therapy	Potentially shorter PFS than BR Increased risk of: ◦ Cytopenia ◦ Myelodysplasia ◦ Secondary myeloid neoplasm
Bortezomib-dexamethasone-rituximab	Ideal for patients who are: ◦ Older/frail ^a^ ◦ Have renal dysfunction ◦ Require rapid disease control ◦ Have MYD88 wild-type or CXCR4 mutations ◦ Prefer time-limited therapy	Increased risk of neuropathy Access varies by province
Rituximab	Ideal for patients who are: ◦ Very frail ^a^ ◦ Have severe cytopenias	Low efficacy compared to other standard therapies Risk of IgM flares, therefore not for patients with hyperviscosity or very high IgM levels
Carfilzomib-dexamethasone-rituximab	Ideal for patients who: ◦ Received previous BR and BTK inhibitor; and ◦ Have bortezomib-associated neuropathy ◦ Fit, without significant cardiac or renal comorbidity	Not approved specifically for WM Access varies by province
Autologous stem cell transplant	Consider in patients who: ◦ Are young and fit ◦ Have received one prior line of therapy ◦ Have severe disease complications (e.g., amyloidosis)	Increased risk of mortality Expertise varies across centres
Purine analogue-based therapy (fludarabine, cladribine)	Consider in patients who: ◦ Are fit ◦ Received previous BR and BTK inhibitor ◦ Cannot tolerate bortezomib-associated neuropathy	Increased risk of myelodysplastic syndrome and secondary malignancies → rarely used

BR, bendamustine-rituximab; BTK, Bruton’s tyrosine kinase; CIT, chemoimmunotherapy; CNS, central nervous system; PFS, progression-free survival; WM, Waldenstrom Macroglobulinemia. ^a^ There is no standard frailty or fitness assessment tool used in clinical practice in Canada for WM. Physician’s determine whether a patient is likely to tolerate therapy based on their own judgment and may include general geriatric assessment tools that take into consideration the comorbidities and functional status of a patient. ^b^ Ibrutinib typically given as monotherapy in Canada. ^c^ Zanubrutinib is indicated for WM at a dose of either 320 mg once-daily or 160 mg twice-daily; however, only the twice-daily dose has been investigated in phase III clinical trials. ^d^ Zanubrutinib is currently not reimbursed by any provinces but is available in all Canadian provinces through a compassionate access program. Provincial reimbursement may vary in the future.

## Data Availability

Not applicable.
